# Feed intake, digestibility and passage kinetics in grazing horses

**DOI:** 10.1038/s41598-026-35647-7

**Published:** 2026-01-22

**Authors:** Martin Bachmann, Mandy Bochnia, Monika Wensch-Dorendorf, Maren Glatter, Stephan Schäfer, Katrin Simroth, Jörg M. Greef, Annette Zeyner

**Affiliations:** 1 Institute of Agricultural and Nutritional Sciences, Martin Luther University Halle-Wittenberg, Halle (Saale), Germany; 2 Institute for Crop and Soil Science, Julius Kühn Institute, Federal Research Centre for Cultivated Plants, Braunschweig, Germany

**Keywords:** Feed intake, Digestibility, Plant markers, *n*-Alkanes, Travelling activity, Foraging, Pasture, Animal behaviour, Animal physiology

## Abstract

Feed intake and digestibility are important indicators for sufficient nutrient supply, but they can be assessed only to a limited extent in horses on pasture. In horses, feed intake is embedded in a complex behavioural pattern of searching, selecting, chewing and almost constant movement called foraging. The objectives of this study were to estimate organic matter intake (OMI) and organic matter digestibility (OMD) in six horses, 24 h/day on pasture, based on plant alkanes and synthetic *n*-hexatriacontane (C_36_) excretion. A multi-compartmental model was fitted to the marker excretion and the C_36_ mean retention time (MRT) was estimated. The travelling activity influences the intestinal passage and digestion of the forage. For this reason, the travelled distances were tracked during the daylight hours (i.e., 13 h/day) by the global positioning system (GPS). The C_36_ MRT was 17.5 h based on an excretion curve, in which all horses were considered. Fitting individual excretion curves was less successful due to limited data points available for each horse. Depending on the plant marker, an OMI of 1.4 to 2.8% of body weight (BW)/day was estimated, which is analogous to a dry matter intake (DMI) of 1.5 to 3.1% of BW/day. The estimated OMD ranged from 0.45 to 0.68, dry matter digestibility (DMD) analogously from 0.39 to 0.65. The estimates obtained using *n*-nonacosane (C_29_), followed by those obtained using *n*-hentriacontane (C_31_), seemed to be the most plausible compared to the literature. Using C_29_, a feed intake of 2.5% of BW/day (group) or 2.1% of BW/day (individuals) was estimated on organic matter basis, which was 2.7% of BW/day or 2.3% of BW/day on dry matter basis. An OMD of 0.64 and a DMD of 0.61 was estimated using C_29_. Individually travelled distances ranged from 0.09 to 4.64 km in 1 h with differences detected among the days (*p* < 0.01), but not among the hours monitored within a day. Despite clear limitations, the methods seemed to be reliable to assess feed intake on pasture and to track movement activity. In modern husbandry systems, foraging should be as much unrestricted as possible to satisfy natural behavioural needs.

## Introduction

Foraging on pasture is an essential behaviour that contributes to the welfare of horses. With free access to pasture, energy and protein can be oversupplied from a nutritional perspective, while the intake of trace minerals is often inadequate^1^. Developmental disorders, obesity, laminitis and other syndromes related to nutrition can be prevented when the intake and the digestibility of energy and nutrients are assessable. In grazing horses, this is methodologically challenging, because balancing of nutrient intake and excretion on the basis of a total collection of faeces is unfeasible. Therefore, reliable marker-based methods are required. Digestibility depends on feed structure and quality and the mean retention time (MRT) in the digestive tract^2^. The MRT can be determined from the excretion of a marker labelling the solid or the liquid phase of digesta, because MRT is defined as the time the marker is retained in the mixing compartments of the digestive tract. In herbivores, several plant markers such as acid insoluble ash, *n*-alkanes, alkanols or fatty acids have been applied to estimate nutrient digestibility from forage or forage-based diets^3–5^. Other than external markers such as chromic oxide or synthetic alkanes, plant markers are less influenced by cyclic inter-day and intra-day variation of their faecal concentrations, which is mainly a consequence of discontinuous marker ingestion^4^. Having steady state conditions in marker excretion is a prerequisite for successful digestibility estimation. The simultaneous use of a plant digestibility marker and a faecal output marker, which is dosed to a known quantity, allows the estimation of feed intake^6^. The combination of a plant alkane or alkanol and a dosed alkane was used commonly to predict forage intake in horses^4–11^. Estimates of faecal output can be obtained from a single dose of an external marker. This procedure enables the simultaneous estimation of intake, digestibility and MRT^12^.

Foraging combines searching, feed selection, chewing and slow-moving activity. Previous studies have shown that there is a great discrepancy between free-ranging horses and stabled horses regarding their foraging time regimen and behaviour^13^. Depending on the season, horses on pasture spend 40 to 60% of the whole day with grazing (i.e., 9 to 16 h)^14–20^. A short-term maximum may occur in early spring or late in summer with approximately 70% of the daytime budget (i.e., 16 to 18 h)^21^. In stabled horses, concentrates in addition to forages reduce the time spend foraging (e.g., a diet consisting of 8 kg hay and 2 kg concentrates requires maximal 6 h/day to be ingested)^22^. The study by Berger et al.^17^ in Przewalski’s horses described a poly-phasic daily feed intake pattern with increased activities during dusk and dawn and a 1.2 to 1.5-fold greater daytime activity. However, a strict distinction of feed intake and other activities leads to a much lower mean intake time (approximately 8 h/day). This highlights the difference between pure feed intake time and the time that considers the entire set of foraging behaviours (containing slow movements during grazing and movements of the head during chewing). Another aspect to consider is the processing of the feeds. Argo et al.^22^ showed pelleting may increase feed intake, and Bochnia et al.^23^ showed different pelleting conditions led to increased speed of ingestion, chewing frequency and saliva production. In this respect, physico-chemical and organoleptic properties of the feed (e.g., hardness or pellet size) affect feed intake patterns. Chewing activity directly influences feed comminution and thus its passage and digestibility. It also influences saliva production, the buffering of gastric acids and the release of agents protective to gastric and gut mucosa. Exercise may reduce MRT of the digesta and this affects nutrient digestibility^24^. On a pasture, this can be applied to the travelling activity of the horses and the distances they travel within a certain time period. The travelling activity likewise differs in horses kept permanently on pasture compared to horses kept temporarily on pasture or just in the stable.

The objectives of the present study were to estimate organic matter intake (OMI), organic matter digestibility (OMD) and MRT of the digesta in horses with 24 h/day pasture access using a set of plant alkanes and a single dose of synthetic *n*-hexatriacontane (C_36_). The distances travelled on pasture during the daylight hours (i.e., 13 h/day) were tracked by the global positioning system (GPS) and evaluated in addition to the marker-based estimations.

## Methods

### Animals

Six mares of Trotter (*n* = 4), Trakehner (*n* = 1) and Mecklenburger breed (*n* = 1) were used in this experiment. The mares´ age ranged from 7 to 14 years. They were neither used for breeding, nor trained for appearance in sports. The initial BW and body condition score (BCS) were 533 ± 41.1 kg and 5.3 ± 0.41 on a 1 to 9 scale^25^, respectively. The mares appertain to the herd of the Research Centre for Agricultural and Nutritional Sciences of the Martin Luther University Halle-Wittenberg. They were not purchased as part of this experiment. The animals were under regular veterinary supervision. No abnormalities related to dental health were observed and no injuries or diseases occurred. Keeping of the horses and the experiment complied with the ARRIVE guidelines. The experiment was carried out in 2015 and, after consultation of the licensing authorities of the Federal State of Saxony-Anhalt, classified as an animal experiment not requiring approval. At that time, the authorities did not assign an animal test number. The experiment was assigned to the internal number P83-15 and was under supervision of the animal welfare officer of the Martin Luther University Halle-Wittenberg.

### Experimental set-up, feeding and sampling

**Fig. 1:**
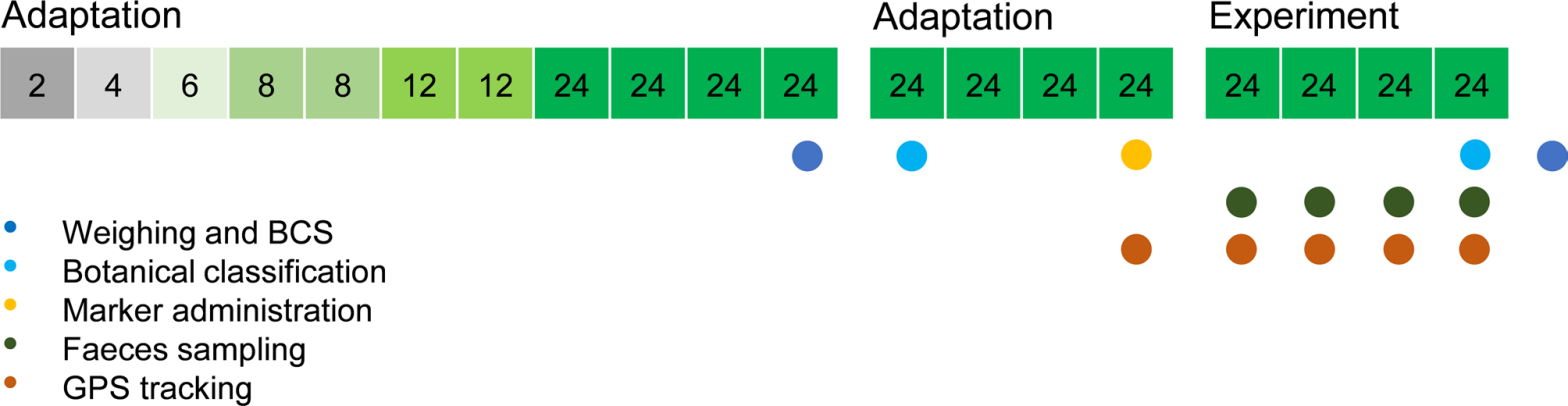
The experiment comprised 11 days of adaptation to pasture, shift to the experimental pasture sward, another 4 days of adaptation and 4 days of faeces sampling. The given numbers display the hours per day the horses were allowed to have pasture access. *BCS* body condition score, *GPS* global positioning system.

Before starting the experiment, the horses were fed meadow hay to meet their maintenance requirements for metabolizable energy (ME) (i.e., 0.52 MJ/kg BW^0.75^) following the recommendations of the Society of Nutrition Physiology (GfE)^26^. They were gradually adapted to the pasture as displayed in Fig. 1. During the first 7 days of adaptation, meadow hay was offered in addition to the pasture. From the eighth day onwards, no additional feeds or any supplements were offered. The horses had *ad libitum* access to fresh water from trough. During the final 4 days of adaptation, placebo variants of the bolus were administered repeatedly by hand to accustom the horses and ensure a high acceptance. The labelled boluses were administered at 7:00 p.m. the last day of adaptation, providing one bolus per individual. The boluses were received without visible losses of the marker or the matrix. During the subsequent 2 days, hand-grab samples were taken of all fresh defecations that occurred between 7:00 a.m. and 9:00 p.m. and stored immediately at -20 °C for subsequent analyses. The 2 days thereafter, maximal five defecations were sampled per day, evenly distributed within the daily examination time. We did not perform a total collection of faeces. The experimental pasture was expanded as needed to ensure a sufficient supply of forage, and to reduce damage of swards by footsteps simultaneously. This led to three subareas used for subsequent sampling.

The experimental pasture subareas were classified for botanical composition, yield proportions and nutritive value before and after the grazing period using the methods of Klapp^27^ and von Boberfeld^28^. Samples of the plant population, their main representatives and concomitant vegetation were taken at four plots (each 1 m^2^) on the subareas 1 and 2, and at three plots on subarea 3, following a diagonal distribution. Plant samples were cut near the ground at approximately 6 cm height. The botanical composition and the yield proportions of the pasture subareas are summarized in Table 1. Before grazing, the plant population was dominated by grass species, where tall oat grass (*Arrhenatherum elatius*), orchard grass (*Dactylis glomerata*), soft brome (*Bromus hordeaceus*), barren brome (*Bromus sterilis*) and quack grass (*Elymus repens*) were identified to be the most abundant ones. The abundance of herbs and legumes was low. However, a distinct vegetation of the large stinging nettle (*Urtica dioica*) was found. In general, swards were dense with maximal 5% gaps and the botanical composition indicated a previous extensive use. The mean nutritive value (classified on a -1 to 8 scale) was 4.7 ± 1.6 on subarea 1, 4.0 ± 0.40 on subarea 2 and 4.2 ± 1.3 on subarea 3, respectively. The pasture was in over-aged maturity with growth heights up to 70 cm. The texture of the soil top layer and the identified vegetation characteristics suggested a humous, sandy to loamy soil substrate. Herbaceous toxic plants were not found on the experimental pasture. However, branches, leaves and seedlings of the sycamore maple tree were accessible in the periphery. The concomitant vegetation of woody plants comprised wild black cherry (*Prunus serotina*), sour cherry (*Prunus cerasus*), lime (*Tilia platyphyllos*), staghorn sumac (*Rhus typhina*) and blackberry (*Rubus fruticosus*), to which the horses had no direct access. After the grazing period, pasture residuals ranged from less than 10 to 20% and mainly comprised *Urtica dioica*. The chemical composition of the pasture is given in Table 2. The alkane concentrations determined in the plant communities, abundant plant species and the top layer soil are given in Table 3.

**Table 1:** Percentage yield proportions of grass, herb and legume species found on the experimental pasture subareas.

	Subarea 1				Subarea 2				Subarea 3		
Plant species	A	B	C	D	A	B	C	D	A	B	C
*A. elatius*	49	20	59	n.e.	n.e.	3	3	25	1	3	2
*D. glomerata*	28	n.e.	5	20	5	3	5	5	55	n.e.	1
*B. hordeaceus*	15	2.5	n.e.	n.e.	0	1	0	0	0	50	5
*B. sterilis*	n.e.	2.5	2	35	5	60	20	4	40	36	72
*E. repens*	2	15	25	25	85	11	70	45	0	n.e.	n.e.
Other grasses^1^	5	0	2	0	0	2	n.e.	n.e.	2	1	0
*U. dioica*	n.e.	60	7	15	5	20	2	20	2	8	17
Other herbs or legumes^1^	1	0	0	5	n.e.	0	0	1	0	2	3

*n.e.* not existing.

^1^Identified species with a yield proportion generally equal or lower than 5% were grasses *Lolium perenne*, *Festuca pratensis*, *Festuca rubra* agg., *Poa annua*, *Poa pratensis*, *Hordeum murinum*, *Agrostis stolonifera* and herbs and legumes *Taraxacum* sect. *Ruderalia* spp., *Galium mollugo*, *Galium aparine*, *Rumex crispus*, *Rumex obtusifolius*, *Minuartia* spp., *Geranium molle*, *Arctium lappa*, *Veronica hederifolia*, *Ceratium holosteoides*, *Trifolium repens*.

**Table 2:** Chemical composition of the pasture subareas, given as arithmetic mean and standard deviation (*n* = 4 in subarea 1 and 2, *n* = 3 in subarea 3).

Item	Subarea 1	Subarea 2	Subarea 3
DM (g/kg)	188 ± 10.4	225 ± 8.73	196 ± 6.02
Crude ash (g/kg DM)	80 ± 6.8	83 ± 7.3	93 ± 4.2
CP (g/kg DM)	178 ± 24.4	162 ± 12.1	124 ± 17.1
Pre-caecal digestible CP (g/kg DM)^1^	93 ± 8.3	74 ± 7.9	52 ± 8.5
Lysine (g/kg DM)	8.8 ± 1.6	7.5 ± 0.26	5.9 ± 0.77
Pre-caecal digestible lysine (g/kg DM)^1^	4.6 ± 0.72	3.5 ± 0.36	2.5 ± 0.37
SAA (g/kg DM)^2^	4.4 ± 0.62	3.8 ± 0.039	3.2 ± 0.40
Pre-caecal digestible SAA (g/kg DM)^1^	2.2 ± 0.25	1.8 ± 0.17	1.3 ± 0.20
Threonine (g/kg DM)	7.8 ± 1.3	7.0 ± 0.076	5.8 ± 0.72
Pre-caecal digestible threonine (g/kg DM)^1^	4.0 ± 0.56	3.3 ± 0.30	2.4 ± 0.34
Tryptophan (g/kg DM)	2.6 ± 0.41	2.2 ± 0.077	1.8 ± 0.31
Acid ether extract (g/kg DM)	25 ± 2.4	22 ± 2.9	19 ± 0.071
Crude fibre (g/kg DM)	301 ± 19.1	312 ± 13.9	306 ± 11.1
aNDFom (g/kg DM)	659 ± 9.53	669 ± 20.7	628 ± 40.7
ADFom (g/kg DM)	336 ± 6.66	348 ± 17.0	355 ± 8.74
ADL (g/kg DM)	31 ± 2.3	36 ± 3.8	35 ± 3.4
Glucose (g/kg DM)	5.9 ± 4.6	4.2 ± 1.7	3.9 ± 1.2
Fructose (g/kg DM)	24 ± 11	33 ± 19	41 ± 11
Mannitol (g/kg DM)	1.8 ± 0.57	0.93 ± 1.2	2.2 ± 0.47
Fructans (g/kg DM)	10 ± 2.0	11 ± 4.4	24 ± 8.0
Gross energy (MJ/kg DM)	18.5 ± 0.399	18.7 ± 0.465	18.0 ± 0.392
ME (MJ/kg DM)^3^	6.9 ± 0.51	6.7 ± 0.31	6.7 ± 0.37
ME (MJ/kg DM)^4^	7.3 ± 0.49	7.0 ± 0.35	6.9 ± 0.30
Phosphorus (g/kg DM)	3.5 ± 0.27	3.2 ± 0.29	2.8 ± 0.27
Calcium (g/kg DM)	4.4 ± 0.93	4.4 ± 0.82	4.8 ± 0.80
Potassium (g/kg DM)	19 ± 3.2	22 ± 2.6	25 ± 2.5
Sodium (g/kg DM)	0.8 ± 0.5	0.2 ± 0.041	0.5 ± 0.087
Magnesium (g/kg DM)	1.6 ± 0.25	1.2 ± 0.19	1.3 ± 0.056
Zinc (mg/kg DM)	22 ± 3.7	23 ± 5.9	21 ± 2.5
Manganese (mg/kg DM)	39 ± 7.5	37 ± 9.3	52 ± 15
Copper (mg/kg DM)	9.5 ± 0.64	9.9 ± 1.4	13.3 ± 1.20
Iron (mg/kg DM)	137 ± 60.9	117 ± 45.6	86 ± 7.1

*AA* amino acids, *ADFom* acid detergent fibre expressed exclusive of residual ash, *ADL* acid detergent lignin, *aNDFom* neutral detergent fibre assayed with amylase and expressed exclusive of residual ash, *CP* crude protein, *DM* dry matter, *ME* metabolizable energy, *SAA* sulphurous AA.

^1^Calculated according to GfE^26^ and Zeyner et al.^29^.

^2^Sum of methionine and cysteine.

^3^Calculated according to GfE^26^ and Kienzle and Zeyner^30^ with static consideration of renal energy losses.

^4^Calculated according to Kuchler et al.^31^ with dynamic consideration of renal energy losses.

**Table 3:** Alkane concentrations in samples of the pasture subareas, main representatives of the plant community and the top layer soil, given as arithmetic mean and standard deviation (*n* = 4 in subarea 1 and 2, *n* = 3 in subarea 3).

Item (mg/kg DM)	C_25_	C_27_	C_29_	C_31_	C_33_
Subarea 1	25 ± 6.6	25 ± 4.9	55 ± 19	63 ± 13	54 ± 8.0
Subarea 2	18 ± 2.0	24 ± 3.0	59 ± 15	64 ± 24	40 ± 5.6
Subarea 3	20 ± 11	34 ± 2.9	105 ± 27.7	108 ± 19.1	42 ± 10
*A. elatius*	11	15	30	48	30
*D. glomerata*	25	20	55	78	36
*B. hordeaceus*	17	23	149	59	14
*B. sterilis*	2	20	112	109	33
*E. repens*	3	4	14	36	42
*U. dioica*	2	8	86	107	5
Soil	0	0	1	2	1

Alkane concentrations of individual plant species and soil are represented by individual samples.

C_25_ = *n*-pentacosane, C_27_ = *n*-heptacosane, C_29_ = *n*-nonacosane, C_31_ = *n*-hentriacontane, C_33_ = *n*-tritriacontane; *DM* dry matter.

Body weights and BCS were measured before permanent pasture access and after the sampling period (Fig. 1). The initial BW of 533 ± 41.1 kg slightly increased to 542 ± 44.1 kg. The BCS mostly remained unaltered and was 5.3 ± 0.41 of 9 before and 5.4 ± 0.33 of 9 after the trial.

### Travelling activity

The horses were equipped with a GPS tracker (POLAR Electro GmbH, Büttelborn, Germany) for individual 13-h time frames each day (7:00 a.m. to 8:00 p.m.) over 5 days to record the travelled distances. To protect the trackers from damage, measurements were not performed during the night hours.

### Preparation of synthetic marker

Synthetic C_36_ (98% purity; Sigma-Aldrich Chemie GmbH, Steinheim, Germany) was prepared as described by Bachmann et al.^32^. Briefly, the total required quantity of C_36_ was melted at around 80 °C in a water bath and then allowed to cool down at room temperature. Afterwards, the recrystallized wax-like form was crushed into small fragments and weighed into hypromellose (HPMC) capsules (size 000; Silvaco A/S, Dah Feng, Taiwan), each one labelled with 750 mg of C_36_. The capsules were finally encased by a pastry made of oat flakes, a sugar beet syrup preparation (ZUEGG Deutschland GmbH, Zörbig, Germany) and wheat flour, mixed by weight 1:0.8:0.6, and a small amount of water. The boluses, 3 cm in diameter, were lyophilized for 24 h. The placebo variants of the boluses were made in a similar manner. The target long-chain alkanes were detected neither in the matrix itself, nor in the HPMC capsules^33^.

### Chemical analyses

Feed and faeces samples were dried at 60 °C or lyophilized for 48 h, respectively, and ground to pass either a 1.0- or 0.5-mm sieve of a sample mill for proximate nutrient analyses or for amino acid and marker analyses, respectively. All feed samples were chopped to coarse particles by machine before processing.

The analyses of dry matter (DM), crude ash (CA), crude protein (CP), acid ether extract (AEE), crude fibre, neutral detergent fibre (aNDFom), acid detergent fibre (ADFom) and acid detergent lignin (ADL) were performed using official methods of the Association of German Agricultural Inspection and Research Institutes (VDLUFA) (methods no. 3.1, 4.1.1, 5.1.1 B, 6.1.1, 6.5.1, 6.5.2, 6.5.3, 8.1)^34^. A FOSS 2300 Kjeltec^™^ Analyzer was used for nitrogen, a FOSS Tecator^™^ Soxtec^™^ 1047 Hydrolyzing and a Soxtec^™^ HT 1043 Extraction unit for AEE and a FOSS FT Fibertec^™^ for crude fibre determination (FOSS GmbH, Rellingen, Germany). The aNDFom was assayed with a heat stable amylase. Neutral detergent fibre and ADFom were expressed exclusive of residual ash. The proteins in the forage samples were hydrolysed with hydrochloric acid and amino acids were analysed using a Biochrom 30 Amino Acid Analyser with PEEK-Sodium Prewash Column (100 × 4.6 mm) and PEEK-Oxidised Feedstuff Column (200 × 4.6 mm) (Biochrom Ltd., Cambridge, UK) according to VDLUFA method no. 4.11.1^34^. Tryptophan was analysed after protein hydrolysis with phosphoric acid and hydrochloric acid using high performance liquid chromatography (HPLC) on an Agilent 1100 Series unit with ZORBAX Eclipse XDB-C8 column (150 × 4.6 mm, 5 μm) (Agilent Technologies Inc., Santa Clara, CA, USA)^35^. Neutral detergent insoluble CP was determined on the basis of the protocol described by Licitra et al.^36^ according to VDLUFA method no. 4.13.1^34^. This was used to calculate pre-caecal digestible CP and pre-caecal digestible amino acids according to Zeyner et al.^29^. Mono- and dimeric sugars (glucose, fructose, mannitol) and fructans were analysed on a KONTRON Instruments HPLC (Tresser Instruments, Rossdorf, Germany) fitted with a Rezex^™^ RPM-Monosaccharide Pb + 2 HPLC column (100 × 7.8 mm) (Phenomenex Ltd. Deutschland, Aschaffenburg, Germany). Gross energy was determined by bomb calorimetry using a C7000 Oxygen Bomb Calorimeter (IKA^®^ Werke, Staufen, Germany). The concentration of ME in the feeds with a static consideration of renal energy losses was calculated according to Kienzle and Zeyner^30^. Additionally, ME was calculated with a dynamic consideration of renal energy losses according to Kuchler et al.^31^. Macro and trace elements were analysed by inductively coupled plasma optical emission spectrometry on a Varian 715-ES ICP-OES (Agilent Technologies Inc., Santa Clara, CA, USA) following extraction as described by Rodehutscord and Dieckmann^37^.

The plant alkanes *n*-pentacosane (C_25_), *n*-heptacosane (C_27_), *n*-nonacosane (C_29_), *n*-hentriacontane (C_31_) and *n*-tritriacontane (C_33_), and the synthetic C_36_ were extracted from feed and faeces and determined by gas chromatography. Briefly, 0.5 g (feed) or 0.25 g (faeces) were saponified in ethanolic potassium hydroxide for 4 h at 90 °C, extracted by phase separation into *n*-heptane at 75 °C and purified through silica-gel columns. Both *n*-docosane and *n*-tetratriacontane (98% purity) were priorly added to each test tube and used as internal standards. Gas chromatography was carried out on a Shimadzu GC-2010 unit fitted with a flame ionization detector (FID) (Shimadzu Corporation, Kyoto, Japan). The target analytes were separated on an Rtx^®^-1 w/Integra-Guard^™^ column (30 m × 0.53 mm, 0.25 μm film thickness) (Restek Corporation, Bellefonte, PA, USA) after on-column injection. The injection volume was 0.5 µL. The injector temperature programme was: 80 °C hold for 0.1 min, raised to 310 °C by 100 K/min, hold for 10 min. The column oven temperature programme was: 80 °C hold for 0.1 min, raised to 240 °C by 50 K/min, hold for 1 min, raised to 264 °C by 6 K/min, to 284 °C by 4 K/min, to 296 °C by 2 K/min, hold for 10 min. Helium was used as the carrier gas at 30.1 cm/s linear velocity (column flow: 3.75 mL/min; pressure: 22.7 kPa). The FID temperature was 315 °C. Helium was the makeup gas with a flow of 30 mL/min. A standard solution containing a homologous sequence of the target alkanes (*n*-docosane to *n*-octatriacontane) was run regularly to identify retention times and to determine a device-internal discrimination of alkanes with increasing molecular weight. Alkane concentrations were quantified on peak area basis in relation to the internal standards and corrected for any discrimination that might have occurred during solvent extraction as described by Oliván and Osoro^38^. To verify dosing accuracy of C_36_ in the boluses and to consider the impact of the melting process, a subsample of C_36_ doses (*n* = 5) was prepared as described above. Each dose was diluted in 10 mL *n*-heptane, then placed in an ultrasonic bath at approximately 40 °C until it was completely dissolved. A proportion of 0.1 mL of each dose was diluted 100-fold in 9.9 mL *n*-heptane and 1 mL of the dilution was used to measure C_36_ by gas chromatography after adding the internal standards.

### Calculations and statistical analysis

Statistical analysis was performed with SAS 9.4. (SAS Institute Inc., Cary, NC, USA). Percentage yield proportions of grass, herb and legume species found on the experimental pasture subareas as well as nutrient and alkane concentrations are reported as descriptive data and did not undergo statistical analysis.

Modelling of the C_36_ excretion curves was performed separately for the group of horses and the individual horses. The multi-compartmental model proposed by Dhanoa et al.^39^ was fitted to the C_36_ excretion curves on the basis of raw measures of the C_36_ concentration using the MODEL procedure:

$$\:{y}_{t}=A\times\:{e}^{{-k}_{1}t}\times\:e\left[-B{e}^{-{k}_{2}t}\right]$$

where *y*_*t*_ is the C_36_ concentration at time *t*, *A* is a scale parameter, *k*_1_ and *k*_2_ are estimates of slow and fast fractional outflow rates (h^−1^), *B* is the number of mixing compartments and *t* is the time after administration of C_36_ (h). The following model parameters were used as seeds on the basis of parameter estimates provided by Bulang et al.^40^: *A* = 28000, *B* = 120, *k*_1_ = 0.12, *k*_2_ = 0.2. For modelling of the C_36_ group curve, data from one horse was removed due to the large deviation of its individual curve from the group curve. Then, a total of 87 individual measures remained for curve modelling. For the modelling of the individual C_36_ curves, data from three horses had to be discarded due to a lack of fitting or implausible parameter estimates and a total of 11 (horse 2), 21 (horse 3) and 15 (horse 6) measures were finally considered. Based on the model’s parameter estimates (*A*, *B*, *k*_1_, *k*_2_), MRT, organic matter output (OMO) and OMI were estimated using the procedure proposed by Giráldez et al.^12^.

The MRT of C_36_ was calculated as:

$$\:\mathrm{MRT}\left(h\right)=\frac{1}{k1}+\frac{1}{k2}+{\sum\:}_{i=3}^{B-1}\frac{1}{k2+\left(i-2\right)\left(k2-k1\right)}$$

where MRT of the mixing compartment with the slow fractional outflow rate (C1) is given as: MRT_C1_ = 1/*k*_1_ and MRT of the mixing compartment with the fast fractional outflow rate (C2) is given as: MRT_C2_ = 1/*k*_2_.

Daily OMO (kg) was estimated on the group scale and for individual horses as (24 × *K* × *D*)/*A*, where *D* is the single dose of C_36_ (mg), *A* is the scale parameter derived from fitting of the multi-compartmental model and *K* was calculated as follows:

$$\:K=k1k2\frac{\langle{\prod\:}_{i=3}^{B-1}\left\{1+k2/\left[\left(i-2\right)\left(k2-k1\right)\right]\right\}\rangle}{\left(B-2\right)\left(k2-k1\right)}$$

The OMI (kg/day) was estimated on the group scale and for individual horses as OMO/(*H*_*i*_/*F*_*i*_), where *H*_*i*_ and *F*_*i*_ are the concentrations (mg/kg organic matter) of the plant alkane *i* in feed and faeces, respectively.

Organic matter digestibility was estimated on the basis of plant alkane concentrations in feed and faeces as 1 – (*H*_*i*_/*F*_*i*_) × (*F*_*n*_/*H*_*n*_), where *H*_*i*_ and *H*_*n*_ are the concentrations of alkane *i* (mg/kg DM) and nutrient *n*, i.e., organic matter (g/kg DM) in feed, and *F*_*i*_ and *F*_*n*_ are the respective concentrations of alkane *i* and nutrient *n* in faeces.

The MRT, OMI, OMD and OMO are given as arithmetic means and the standard deviation is reported. Faecal recovery was not considered for correction of the measured alkane concentrations.

The travelled distances were analysed using the MIXED procedure and the following linear model: *y*_*ijk*_ *= µ + α*_*i*_ *+ β*_*j*_ *+* *a*_*k*_ *+* *ε*_*ijk*_

where *y*_*ijk*_ is the travelled distance, *µ* is the general mean, *α*_*i*_ is the fixed effect of day *i* (*i* = 1, …, 5), *β*_*j*_ is the fixed effect of hour *j* (*j* = 1, …, 13), *a*_*k*_ is the random effect of animal *k* (*k* = 1, …, 5), considering repeated measurements per animal, and *ε*_*ijk*_ is the random residual effect with *ε*_*ijk*_ ~ N(0,σ^2^*ε*). From one horse, GPS data was not available. For the travelled distances, arithmetic means are given and the standard deviation is reported.

The significance level for the statistical tests was set to *p* < 0.05.

## Results and discussion

### Mean retention time and faecal output

**Fig. 2:**
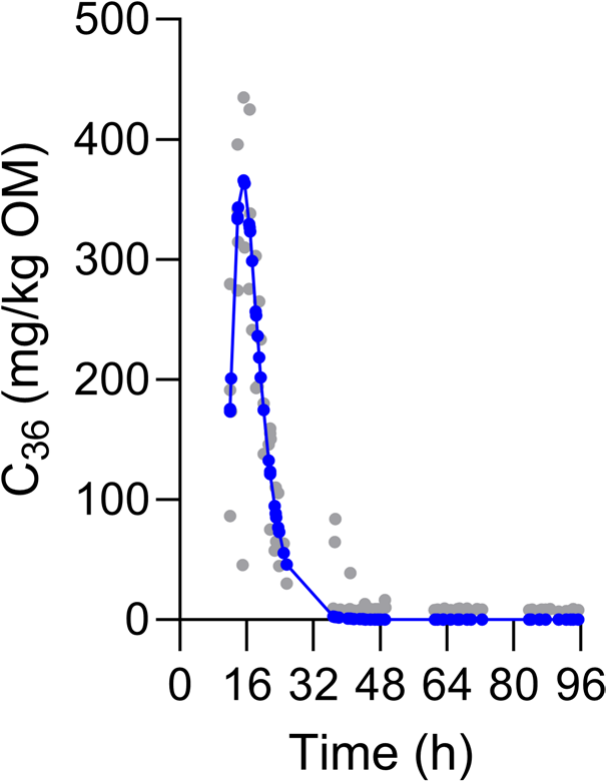
Faecal excretion curve of *n*-hexatriacontane (C_36_) shown for the group of five horses. The curve was obtained from fitting the multi-compartmental model of Dhanoa et al.^39^ (blue dots) to individual measures of C_36_ faecal concentration (grey dots). C_36_ was administered at time point 0 as a single dose of 629 ± 147 mg. *OM* organic matter.

The applied single dose of C_36_ was 629 ± 147 mg based on a subsample of five equally produced bolus doses. The multi-compartmental model sufficiently fitted C_36_ excretion for the group of horses (*R*^2^ = 0.93). The estimates of the model parameters were as follows: *A* = 31105.4, *B* = 310.4, *k*_1_ = 0.254 h^−1^, *k*_2_ = 0.668 h^−1^. The faecal excretion curve is displayed in Fig. 2. Curve fitting of individual horses’ C_36_ excretion and parameter estimation was successful for only three out of six animals, in which *R*^2^ ≥ 0.95. The kinetics parameters both estimated from the group curve and from individual C_36_ excretion curves are given in Table 4. On that basis, the OMO was estimated 4.7 kg/day for the group and 3.9 ± 0.19 kg/day for the individual horses.

**Table 4:** Kinetics parameter estimates derived from the group or individual *n*-hexatriacontane (C_36_) excretion curves.

Kinetics parameter (h)	Group (*n* = 5)	Individuals (*n* = 3)
MRT_C1_	3.9	5.6 ± 2.7
MRT_C2_	1.5	1.4 ± 0.22
Total MRT	17.5	18.5 ± 2.17

Estimates based on the individual curves are given as arithmetic mean and standard deviation.

*C1* mixing compartment with slow fractional outflow rate, *C2* mixing compartment with fast fractional outflow rate, *MRT* mean retention time.

The concentrations of the plant alkanes we used as markers were in a range reported in literature^41,42^. Alkanes are part of the epicuticular and intra-cuticular waxes and therefore they are part of the plants’ defence strategy against drought, irradiation, pollutants, parasites and predators. Alkane concentrations may largely vary among plant species, habitats, plant organs or maturity stages^42^. The large variability of the alkane concentrations within the complex plant community of a pasture may limit the reliability of the estimates obtained using such substances as digestibility markers. Selective feed intake may further complicate these estimates. However, all available markers have uncertainties, which is why their use generally provides only a rough orientation.

For the mathematical fitting of the C_36_ excretion curves, the G3 and G4 models of Pond et al.^43^ and the multi-compartmental model of Dhanoa et al.^39^ were tested, because these models were previously recommended for the use in horses on forage-based diets^44,45^. However, only the multi-compartmental model has shown complete convergence and adequate fitting with *R*^2^ values greater than 0.93. This confirms with the results of Miyaij et al.^46^. Fitting the C_36_ group curve was more successful than fitting curves of C_36_ excretion by individual animals due to the larger data base.

The obtained MRT_C1_ (3.9 h), MRT_C2_ (1.5 h) and total MRT (17.5 h) were lower than values reported in literature based on feeding of freshly cut forage (MRT was 23 to 32 h using polyethylene pieces)^47^, *ad libitum* hay (MRT_C1_ was 5.4 to 6.6 h, MRT_C2_ was 2.4 to 2.9 h and total MRT was 23 to 25 h using chromium-mordanted hay)^46^ or hay offered at restricted quantities (MRT_C1_ was 6.7 to 15.6 h, MRT_C2_ was 3.2 to 9.4 h and total MRT was 25 to 29 h using chromium-mordanted or ytterbium-labelled hay)^45,46^. According to Miyaij et al.^46^, C1 refers to the compartment, which has the longest MRT, and C2 to the compartment, which has the second longest MRT. In horses, this might reflect MRT of the colon (C1) and the caecum (C2), but it is generally difficult to translate a mathematical construct to real anatomical structures or physiological conditions^46^. Mean retention time is affected by the feed intake level^48^, diet composition (i.e., forage-to-concentrate ratio)^24^, forage particle size distribution and forage quality^46^, exercise^24,49^, the model used for data fitting^44,45^ and probably by the marker itself. Depending on the labelling technique and marker administration form, externally applied markers may differentially pass the digestive tract and migrate between solids and the liquid phase^40^. In this study, GPS tracking revealed that the individual horses travelled between 0.09 and 4.64 km in an hour during the daytime period. If the horses stood still during this time, this was not recorded and eliminated. Thus, compared to horses in a stable, accelerated digesta passage on pasture is mainly an effect of increased travelling activity (an exercise effect). Faecal output on pasture was similar to the estimates given, e.g., by Chavez et al.^50^.

### Organic matter intake and digestibility

Organic matter intake and OMD estimated on pasture are given in Table 5, distinguished for the applied markers. The OMI ranged from 7.5 to 14.9 kg/day, which can be expressed as 1.4 to 2.8% of BW/day or 1.5 to 3.1% of BW/day on DM basis.

**Table 5:** Estimates of organic matter intake (OMI) based on faecal output, which derived from the group or individual *n*-hexatriacontane (C_36_) excretion and different digestibility markers, and organic matter digestibility (OMD) based on these markers.

Marker	OMI (kg/day)	OMD
*n*-pentacosane (C_25_)	9.1 / 7.9 ± 1.4	0.47 ± 0.060
*n*-heptacosane (C_27_)	9.6 / 8.0 ± 1.7	0.50 ± 0.072
*n*-nonacosane (C_29_)	13.4 / 11.4 ± 0.93	0.64 ± 0.032
*n*-hentriacontane (C_31_)	14.9 / 12.6 ± 0.89	0.68 ± 0.023
*n*-tritriacontane (C_33_)	8.7 / 7.5 ± 0.51	0.45 ± 0.023

The OMD is given as coefficients. Estimates based on C_36_ excretion of the group of horses (*n* = 5) are given before the slash, estimates based on individual C_36_ excretion (*n* = 3) are given as arithmetic mean and standard deviation thereafter.

The estimated OMI was in line with Friend et al.^10^, who found OMI in grazing weanlings (fillies with 355 kg mean BW and colts with 266 kg mean BW) between 3 and 11 kg/day, and in line with Chavez et al.^50^, who found dry matter intake (DMI) of 2.7% of BW/day (C_36_/C_31_ or C_36_/C_33_) in adult horses (568 kg mean BW) with daily 12 h pasture access. The latter was similar to the estimates we made with C_36_/C_29_ on group (2.7% of BW/day) and C_36_/C_29_ (2.3% of BW/day) or C_36_/C_31_ (2.6% of BW/day) on an individual scale. The model provided by Siciliano (i.e., intake as g DM/kg BW = 5.12 × √24 – 2.86 with 24 h pasture access)^51^ predicted a DMI of 11.8 kg/day or 2.2% of BW/day. The best match of this estimation was with the individual estimates we obtained using C_36_/C_29_ as the marker pair; i.e., DMI of 12.5 kg/day in horse 3, 11.4 kg/day in horse 4 and 13.4 kg/day in horse 6. On a group scale, C_36_/C_27_ was the marker pair that best matched the DMI estimation of Siciliano^51^; i.e., 10.5 vs. 11.8 kg/day. Fernandes et al.^47^ determined 65 g DM of freshly cut forage ingested per kg BW^0.75^, which is 7.2 kg/day in the horses in this study, and this is similar to DMI estimates we obtained from an indoor trial, in which the horses received freshly cut forage, with C_36_/C_29_ as marker pair on the group scale (7 kg/day) or C_36_/_31_ in individual horses (7 kg/day) (unpublished data). Reduced feed intake in the stable is plausible, because moving ability is restricted and MRT of solid digesta delayed.

Chavez et al.^50^ reported DMD of 0.61 (C_31_) or 0.60 (C_33_) on pasture with restricted access, Martin-Rosset et al.^52^ an OMD of 0.66 and DMD of 0.63 and Smolders et al.^53^ an OMD of 0.72 with freshly cut forage. These coefficients were very similar to those we obtained with C_29_ (0.64 OMD and 0.61 DMD) and C_31_ (0.68 OMD and 0.65 DMD). The pasture quality was widely comparable to previous studies, e.g., Chavez et al.^50^ or Smolders et al.^53^, similar in CP (155 g/kg DM in relation to 139 to 156 g/kg DM), but higher in aNDFom (652 g/kg DM in relation to 450 to 561 g/kg DM).

Faecal recovery of long-chain *n*-alkanes in grazing horses were reported in a range of 0.86 to 1.04 (C_27_), 0.88 to 0.99 (C_29_), 0.87 to 1.02 (C_31_) and 0.84 to 1.08 (C_33_)^54^. Ribeiro et al.^55^ reported an average faecal recovery of C_36_ of 0.92 with hay. The horses used in our study had a mean faecal recovery of C_36_ of 0.97 when fed fresh forage (unpublished). To estimate faecal output, incomplete faecal recovery is not a problem if it is constant and for intake estimation, it is not a problem if it is equal in the plant and dosed alkane^12^. Plant alkane concentration can be very variable^42^. Thus, this concept is often not applicable. Failure to consider faecal marker recovery can lead to errors in OMI, OMD and OMO estimation. However, determining recovery rates on pasture is not feasible as it would require a total collection of faeces.

### Travelling activity

**Fig. 3:**
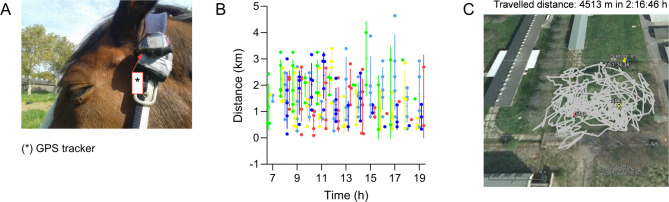
(**A**) Illustration of the GPS tracker position. (**B**) Travelling activity pattern over the 13-h measuring period for 5 days. Colour legend: green day 1, yellow day 2, light blue day 3, dark blue day 4, red day 5. The lines indicate the arithmetic mean and standard deviation. (**C**) Exemplary illustration of GPS tracking of one horse on pasture over a 2-h period. The time at start was 2015-05-18 9.01 a.m., the duration of measurement was 2:16:46 h and the travelled distance within this time frame was 4513 m. Background satellite image: © 2015 Google, © 2009 GeoBasis-DE/BKG, Image © 2015 GeoBasis-DE/BKG. *GPS* global positioning system.

The used equipment for tracking the travelled distances is illustrated in Fig. 3A. Over the time of measurement (7:00 a.m. to 8:00 p.m.), the horses showed nearly non-stop grazing. The distances covered by the horses within the 13-h measuring period were 1.98 ± 0.862 km in 1 h (day 1), 1.37 ± 0.865 km in 1 h (day 2), 1.61 ± 0.938 km in 1 h (day 3), 1.43 ± 0.871 km in 1 h (day 4) and 1.33 ± 0.864 km in 1 h (day 5). The travelled distances differed among the days (*p <* 0.01), but did not differ among the hours measured within a day. The travelling activity pattern is shown in Fig. 3B for the consecutive 5 days. An exemplary data visualization of the GPS tracking of one horse is provided in Fig. 3C. During data collection, the horses remained on the same pasture area and no further subarea was added.

The travelled distances showed a high motivation for walking as part of the foraging behaviour, although the available pasture area was limited to 0.5 ha in total. The local and experimental conditions did not enable to provide a larger area. For a number of six horses, 4.3 ha pasture are the recommended stocking density^56^. The travelled distances we measured were in contrast to a study conducted by Schmitz et al.^57^ with a citizen science approach, where horses had walking distances of only 500 m in 1 h. This is only a third or quarter of the distances observed in the present study. The pasture size was similar, at least 0.2 ha for rotational grazing and 0.5 ha for continuous grazing, but pasture access was restricted. However, the study of Hampson et al.^58^ illustraded that the daily distance travelled might be expressed as a function of increasing paddock or pasture size. Domesticated horses increased the distance from 1.1 km/day on a 6 × 6 m paddock to 7.2 km/day on 16 ha^58^. In contrast, feral horses on 4000 ha travelled 18 km/day^58^. Our observations have shown that horses adapted to 24-h pasture showed a calm and consistent feed intake behaviour and long walking distances in the group.

## Conclusions

Mathematical models can be applied to describe the excretion of an external marker and its passage kinetics. The simultaneous application of *n*-alkanes or other plant markers enables estimation of feed intake and digestibility. The results of this preliminary study suggest this method can be reliable in grazing horses. The reported results must be interpreted and used with caution due to the limited number of horses and samples. The multi-compartmental model showed sufficient convergence when all horses of a group were considered together. The estimation of individual excretion curves was less successful and is not recommended. The most reliable estimates of OMI and OMD were obtained using C_29_ as marker. The observations we made in addition to GPS tracking confirmed continuous foraging on pasture with steady reposeful locomotion. This suggests that unrestricted grazing in a group is closest to the natural behaviour of horses and should be considered in husbandry concepts whenever possible.

## Data Availability

The datasets generated during and/or analysed during the current study are available from the corresponding author on reasonable request.
